# Indian higher education and youth mental health: Challenges and opportunities

**DOI:** 10.7189/jogh.10.020307

**Published:** 2020-12

**Authors:** Seema Mehrotra

**Affiliations:** National Institute of Mental Health and Neurosciences (NIMHANS), Bengaluru, India

Adolescence and young adulthood are globally recognized as periods marked by opportunities for development and the emergence of mental health vulnerabilities [1,2]. The high prevalence of common mental health problems during adolescence and young adulthood, the substantial treatment gap, and multiple barriers to help-seeking at this age highlight the need for comprehensive, campus-based youth mental health approaches grounded in sociocultural realities [3-5].

India has one of the largest higher education systems globally, as well as a high proportion of youth, demographically [6,7]. According to the 2018-2019 All India Survey on Higher Education, there are 993 universities and an average of 28 colleges per lakh of the eligible youth population. About 37.4 million youth are enrolled in institutes of higher education in India. This scenario presents both opportunities and challenges for addressing youth mental health needs [6].

## CHALLENGES

The 2014 Indian National Youth Policy incorporates objectives such as developing a healthy generation and promoting community service [7]. Several governmental youth development programs (eg, Nehru Yuva Kendra Sangathan, National Service Scheme) promote leadership qualities and administration abilities. Engaging young people and faculty in national concerns along with the provision of opportunities for community service are advocated in the draft of the 2019 National Educational Policy in India, which laments the current rigid boundaries of disciplines, an exam-centric approach, and inadequate psychological support for students in higher education [8]. This new policy and the University Grants Commission (UGC) guidelines indicate that student counseling systems must be in place in institutes of higher education (IHE), and teacher-counselors need to be trained to offer psychological support to youth [9]. Also, the UGC recommends setting up student counseling centers. However, there have been gaps in the implementation of relevant policies and programs. Unfortunately, there are no data on the uniform implementation of such guidelines across IHE. Field observations suggest that, while several institutes have made provisions for counseling services in some way, there are no systems in place to continuously capture the nature and quality of services offered, nor do they gather and assess student perspectives on ease of access and utility. In several instances, counseling services when available tend to have suboptimal uptake.

A continuum approach to mental health care is needed—an approach that includes promotive and preventive universal programs for all college youth, along with targeted intervention options for those experiencing significant distress and mental health problems. Such a system should offer targeted interventions across a spectrum of intensity, starting with structured self-help modules for common mental health concerns, peer support systems, and mentor support for mental health in addition to easily accessible counseling services, and timely referrals to specialized mental health professional services. Currently, access to such a range of interventions is highly variable across institutes. Although there are UGC guidelines for the inclusion of youth development components in student induction and mentoring sessions, the uniform inclusion of a standardized training module on mentoring for mental health is needed for faculty in IHE.

Most importantly, the mere availability of counseling services would be insufficient for ensuring uptake and desired mental health outcomes. Apart from reducing scarcity in the supply of resources (eg, trained personnel), the system should foster innovations to handle demand-side barriers (eg, perceived low need and stigma) [10]. Even when multilevel services are available for youth in distress, the services are unlikely to be used unless they are embedded in an enabling environment that helps break psychosocial barriers to help-seeking and promotes a positive mental health culture on campus.

## OPPORTUNITIES

An environment that promotes mental health care can be facilitated through youth engagement programs focused on mental health themes. Initiatives that engage youth volunteers, by familiarizing them with the continuum approach to mental health and inspire those youth to carry out activities that destigmatize mental health and professional help-seeking amongst peers can play an important role [11]. Youth volunteers could also be trained to serve as gatekeepers and first-line support providers. While such youth-driven initiatives should engage youth across varied streams of study, these could be particularly useful for students in the field of psychology and related disciplines. Many colleges have students enrolled in psychology courses who could benefit from such training and inclusion of action projects on mental health promotion and peer support in their curricula. In addition to addressing unmet youth mental health needs, this strategy can also provide students of psychology a hands-on experience in applying their knowledge in a real-world context, allowing them to work with peers on challenges such as stigma and the hidden burdens related to mental health.

**Figure:**
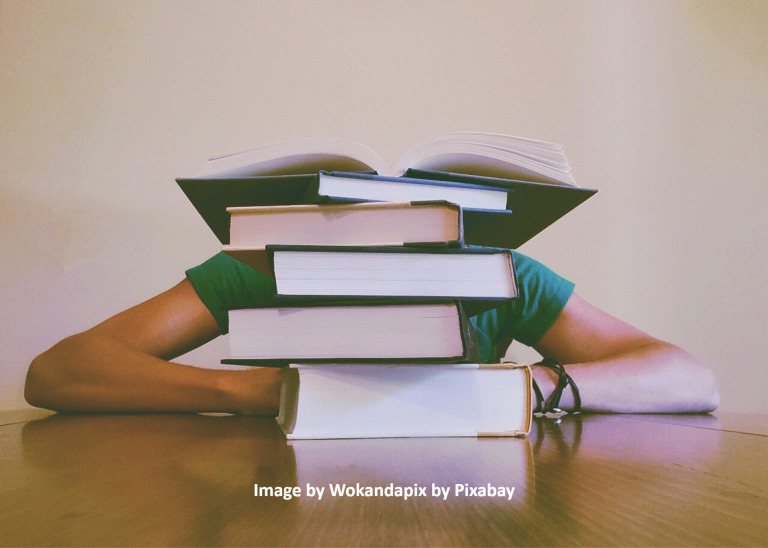
Photo: https://pixabay.com/photos/books-student-studying-learn-read-927394/.

There is a severe shortage of trained mental health resources in India; this shortage partially drives the insufficient engagement of professionals in systematic efforts towards de-stigmatization of mental health and promotive interventions. Large-scale, uniform implementation of youth engagement for mental health initiatives across IHE could be a cost-effective way to address this gap. The 2019 National Educational Policy recommends that, to the extent possible, youth engagement in community service should be integrated within the curriculum, and the time allotted for social engagement for each student should be at least equal to a full semester across the duration of the course [8]. This provides a big window of opportunity for training volunteer-youth for mental health promotion on campus.

Counselors and interested faculty could be trained to set up mental health promotion systems on campuses and bring about systemic changes rather than limiting their role to offering one-to-one counseling services to those who approach or are referred to them. Field observations suggest that universal promotive intervention programs are likely to be well received by college youth when offered on youth-centric themes and involve choice-based enrollment. Interested faculty could be trained using a “training of trainers” cascade model to provide brief promotive modules on a predetermined set of mental health themes. Promotive interventions strengthen protective factors and resilience, and they serve as gateways for youth to negotiate professional help-seeking for mental health [10].

## PATHWAYS TO THE FUTURE- IMPLICATIONS FOR IMPLEMENTATION

A cursory scan of the global initiatives for youth mental health reveals massive funding support across nations for large-scale implementation and continuous monitoring. In India, although there is a growing recognition of youth mental health needs by policymakers, faculty, and administrators; the will to invest in broad-based mental health initiatives within campuses on a sustained basis is considerably weak, partially due to perceived limitations in feasibility, a lack of conviction, and competing academics and other programs [12]. Specific mandates and support from regulatory bodies, advocacy efforts by mental health professionals, and incentivization of such efforts by institutes can help address this situation. There is a need for advocacy and sensitizing of stakeholders in the higher education system in India for making space to implement the frameworks that legitimize and prioritize multipronged mental health promotion systems for college youth. Active involvement of mental health professionals is required in garnering support and buy-in from the educational sector. This can be achieved through synthesizing data available from various countries on the psychosocial and economic benefits of investing in youth mental health and the different ways in which such programs are being implemented globally in IHE.

The digital pathway is another underutilized route to address unmet mental health needs in Indian IHE. Despite good internet penetration, high engagement of youth in online platforms, and the potential flexibility and appeal of digital health tools, there is a scarcity of research-backed mental health tools developed by or in collaboration with mental health professionals in India [13]. Their scalability and acceptability potential are tremendous and need to be optimally utilized. A youth-centric, comprehensive digital platform across IHE can serve as an essential resource for youth who may be exploring mental health information and care options for themselves and others. This could be a multifunction platform accessible to all youth in IHE that provides information, resources, credible links to self-help material, and opportunities to connect to trained peer supporters and counselors online and gives access to a directory of professional services. These tools can also serve as channels for de-stigmatization and positive shifts in perceived peer norms related to professional help-seeking.

## CONCLUSIONS

In low resource contexts, the utilization and mobilization of existing support structures in educational institutes can enhance feasibility, receptivity, scalability, and sustainability of promotive programs for youth. Youth engagement in mental health initiatives, universal promotive interventions, and the optimum use of digital platforms are interlinked, underutilized, and scalable approaches to campus mental health promotion in India. These would be in alignment with the visions of the national youth policy and the recent draft of national education policy in India. These have the potential to reduce the wide treatment gap for common mental health problems as well as empower youth and campus communities to promote youth mental health in general.
